# Subinhibitory Concentrations of Allicin Decrease Uropathogenic *Escherichia coli* (UPEC) Biofilm Formation, Adhesion Ability, and Swimming Motility

**DOI:** 10.3390/ijms17070979

**Published:** 2016-06-29

**Authors:** Xiaolong Yang, Kaihui Sha, Guangya Xu, Hanwen Tian, Xiaoying Wang, Shanze Chen, Yi Wang, Jingyu Li, Junli Chen, Ning Huang

**Affiliations:** Research Unit of Infection and Immunity, Department of Pathophysiology, West China School of Preclinical & Forensic Medicine, Sichuan University, Chengdu 610000, Sichuan, China; fantasyyxl@sina.com (X.Y.); shakaihui@scu.edu.cn (K.S.); xuguangya@scu.edu.cn (G.X.); tianhanwen@scu.edu.cn (H.T.); wangxiaoying@scu.edu.cn (X.W.); chenshanze@scu.edu.cn (S.C.); wy4504228@163.com (Y.W.); jingyuli@scu.edu.cn (J.L.)

**Keywords:** allicin, uropathogenic *Escherichia coli*, biofilm formation and dispersal

## Abstract

Uropathogenic *Escherichia coli* (UPEC) biofilm formation enables the organism to avoid the host immune system, resist antibiotics, and provide a reservoir for persistent infection. Once the biofilm is established, eradication of the infection becomes difficult. Therefore, strategies against UPEC biofilm are urgently required. In this study, we investigated the effect of allicin, isolated from garlic essential oil, on UPEC CFT073 and J96 biofilm formation and dispersal, along with its effect on UPEC adhesion ability and swimming motility. Sub-inhibitory concentrations (sub-MICs) of allicin decreased UPEC biofilm formation and affected its architecture. Allicin was also capable of dispersing biofilm. Furthermore, allicin decreased the bacterial adhesion ability and swimming motility, which are important for biofilm formation. Real-time quantitative polymerase chain reaction (RT-qPCR) revealed that allicin decreased the expression of UPEC type 1 fimbriae adhesin gene *fimH*. Docking studies suggested that allicin was located within the binding pocket of heptyl α-d-mannopyrannoside in FimH and formed hydrogen bonds with Phe1 and Asn135. In addition, allicin decreased the expression of the two-component regulatory systems (TCSs) cognate response regulator gene *uvrY* and increased the expression of the RNA binding global regulatory protein gene *csrA* of UPEC CFT073, which is associated with UPEC biofilm. The findings suggest that sub-MICs of allicin are capable of affecting UPEC biofilm formation and dispersal, and decreasing UPEC adhesion ability and swimming motility.

## 1. Introduction

Urinary tract infections (UTIs) are one of the most common infections both in the community and in hospitals. Uropathogenic *Escherichia coli* (UPEC) are the major cause of uncomplicated UTIs and can cause complicated UTIs. UPEC contaminate the periurethral area and colonize the urethra. Subsequent migration to the bladder enables UPEC to colonize and invade superficial umbrella cells. In the bladder, the UPEC evolve a number of strategies against the host defense system, including biofilm formation [1]. Biofilm protects UPEC from the host immune system, antibiotics, and provides a reservoir for persistent infection. UPEC can also form biofilms on catheters, which lead to catheter-associated urinary tract infections (CAUTIs) [2]. Therefore, UPEC biofilm makes UTIs difficult to eradicate.

To establish a biofilm, the first step of UPEC is to adhere to uroepithelial cells or catheter surfaces, which are mediated by type 1 pili. Therefore, type 1 pili adhesin FimH and its encoding gene *fimH* can be associated with UPEC biofilm formation. Then, the motility of UPEC enables organisms to migrate around facilitating biofilm expansion [3]. In addition, adhesion ability and motility are important for virulence and pathogenicity of UPEC. Two-component systems (TCSs) are ubiquitous signaling units found in prokaryotes which consist of a sensor histidine kinase and a response regulator, and were found to be key mediator of bacterial signal transduction. It has been reported that TCSs were involved in UPEC biofilm formation by sensing various environmental stimulation and regulating gene expression in UPEC [4,5,6]. For instance, the BarA-UvrY, as a TCS in *Escherichia coli* (*E. coli*), is associated with the regulation of several metabolic processes [7,8]. It has been demonstrated that CsrA protein, a component of carbon storage regulatory system, is regulated by BarA-UvrY TCS and it can directly target several genes which are involved in biofilm formation [8]. In addition, loss of *uvrY* in CFT073 can significantly reduce the bacterial attachment to abiotic surfaces and reduce motility in CFT073 [4]. Thus, the BarA/UvrY/Csr pathway plays a critical role in UPEC biofilm formation [4].

**Figure 1:**
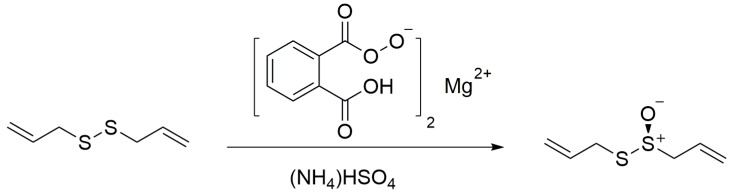
Synthesis of allicin.

It is noticeable that plant-derived antimicrobial agents are active against pathogens, making them promising “antibiotic potentiators” or “virulence attenuators” for the control of infectious diseases [9]. For instance, garlic-derived compounds have shown the ability to inhibit bacteria pathogenicity [10]. Allicin (diallylthiosulfinate, shown in Figure 1), one of the essential oils isolated from garlic (*Allium sativum* L.), has been used in antibacterial and antiseptic therapeutics since World War I [11]. Recently, sub-inhibitory concentrations (sub-MICs) of allicin have been shown to inhibit the formation of *Candida albicans* biofilm [12] and *Staphylococcus epidermidis* biofilm [13], whereas the underlying mechanisms are still not well understood. In this study, we investigated the inhibitory effect of sub-MICs of allicin on UPEC CFT073 and J96 biofilm formation and dispersal. Swimming motility of UPEC was also decreased by allicin. Subsequently, we demonstrated that allicin was essential for decreasing UPEC adhesion ability to T24 cells which might be due to its influence on UPEC adhesin FimH. Influence on gene expression of uvrY and csrA showed that the regulation of the BarA/UvrY/Csr pathway might also be a probable mechanism of antibiofilm activity of allicin.

## 2. Results

### 2.1. The Synthesis of Allicin

The allicin (purity HPLC 96%) used in this study was synthesized by oxidation of diallyldisulphide with magnesium monoperoxyphthalate using tetrabutyl ammonium hydrogensulfate as phase transfer catalyst (yield: 60%) (Figure 1). For the structural characterization, ^1^H-NMR and ^13^C-NMR were used (Figure S1).

### 2.2. The Effect of Allicin on Uropathogenic Escherichia coli (UPEC) Growth, Biofilm Formation and Dispersal

**Figure 2:**
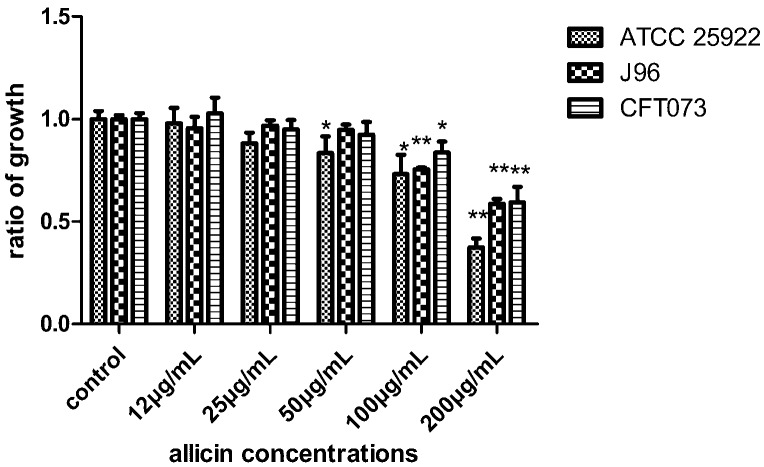
The growth of ATCC 25922, uropathogenic *Escherichia coli* (UPEC) J96, and CFT073 was evaluated by measuring optical density (OD) value at 600 nm after 24 h exposure to allicin and represented as growth ratio relative to control, set at 1. Bars indicate means ± SD of three independent experiments performed in triplicate, * *p* < 0.05, ** *p* < 0.01.

**Figure 3:**
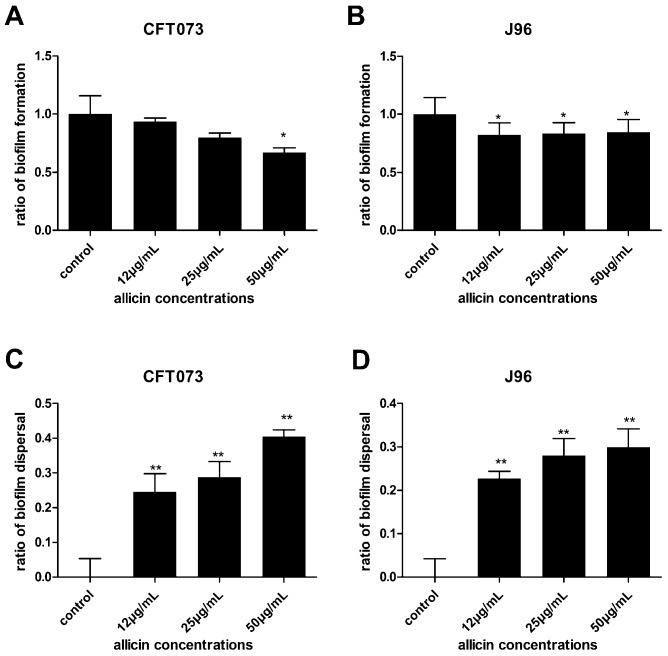
The effect of sub-inhibitory concentrations (sub-MICs) of allicin on UPEC biofilm formation (**A**,**B**) and dispersal (**C**,**D**). The biofilm values were normalized with respect to growth values (**A**,**B**). The biofilm formation was evaluated by measuring OD value at 595 nm and represented as ratio of biofilm formation relative to control, set at 1. Ratio of biofilm dispersal = (OD value of control − OD value of allicin treatment)/OD value of control. Values shown denote the mean ± SD of three independent experiments in triplicate, * and ** mean statistically significant differences compared to control with values *p* < 0.05 and *p* < 0.01, respectively.

UPEC CFT073 and J96 were grown in LB broth in the presence and absence of allicin. Concentrations above 100 µg/mL of allicin slightly decreased the growth of UPEC CFT073 and J96 (Figure 2). A similar trend was observed for *E. coli* ATCC 25922 (Figure 2). Concentrations of allicin of 12, 25 and 50 µg/mL were chosen for subsequent experiments since their effect on the growth of UPEC was negligible. Moreover, the biofilm values were normalized with respect to growth values as described previously [14]. The UPEC biofilm levels were presented in Figure 3 for both biofilms pre-treated or post-treated with allicin. In the case of allicin pre-treatment, UPEC CFT073 and J96 formed less biofilm when incubated with allicin. 50 µg/mL allicin decreased CFT073 biofilm by 33% compared to the control (Figure 3A) while J96 biofilm formation was decreased approximately 17% by all concentrations of allicin tested (Figure 3B). Furthermore, all concentrations of allicin tested dispersed the biofilm significantly. CFT073 biofilm dispersed approximately 40% compared to the control (untreated biofilm) (Figure 3C) and 30% for J96 biofilm (Figure 3D) when post-treated with 50 µg/mL of allicin for 1 h. Thus, allicin can inhibit UPEC biofilm formation and promote their dispersal.

### 2.3. The Effect of Allicin on UPEC Biofilm Architecture

**Figure 4:**
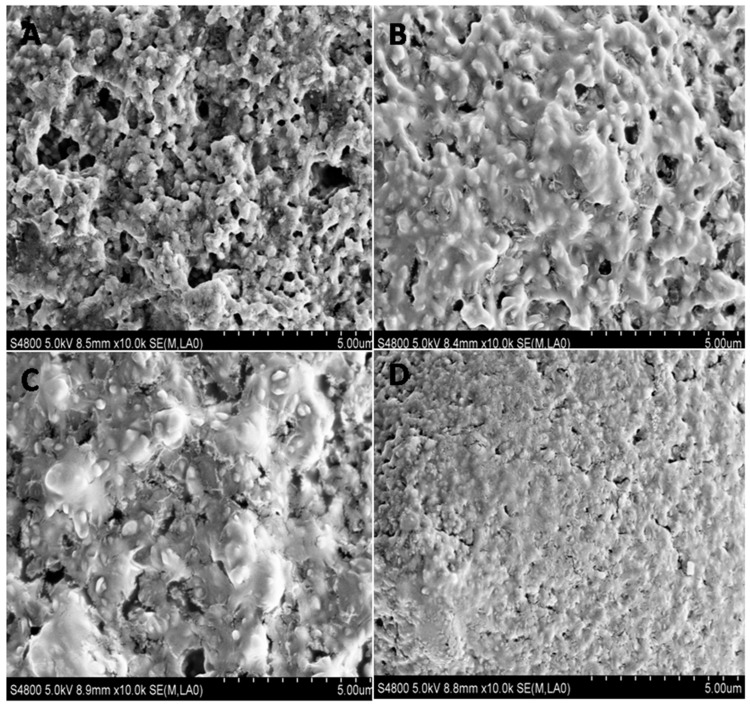
Scanning electron microscopy pictures of growing UPEC CFT073 biofilm. Biofilm architecture was investigated via SEM in the presence or absence of allicin. The images were untreated control (**A**) or treated with 12 µg/mL (**B**); 25 µg/mL (**C**); and 50 µg/mL (**D**) of allicin, respectively.

The impact of allicin on UPEC biofilm architecture was investigated via scanning electron microscopy (SEM). UPEC CFT073 biofilms were dense multicellular communities embedded in an extracellular matrix and the surface was rough with channels inside (Figure 4A). After 24 h of exposure to all concentrations of allicin tested, biofilms formed by CFT073 altered in matrix architecture. The biofilm exhibited mucoid and smooth phenotype and the channels were covered by mucus (Figure 4B). The effects were more obvious when higher concentrations of allicin were applied (Figure 4C,D). UPEC J96 biofilm showed a similar phenomenon (Figure S2). Therefore, the UPEC biofilm architecture was changed in the presence of allicin.

### 2.4. Inhibition of Allicin on UPEC Adhesion Ability and Swimming Motility

**Figure 5:**
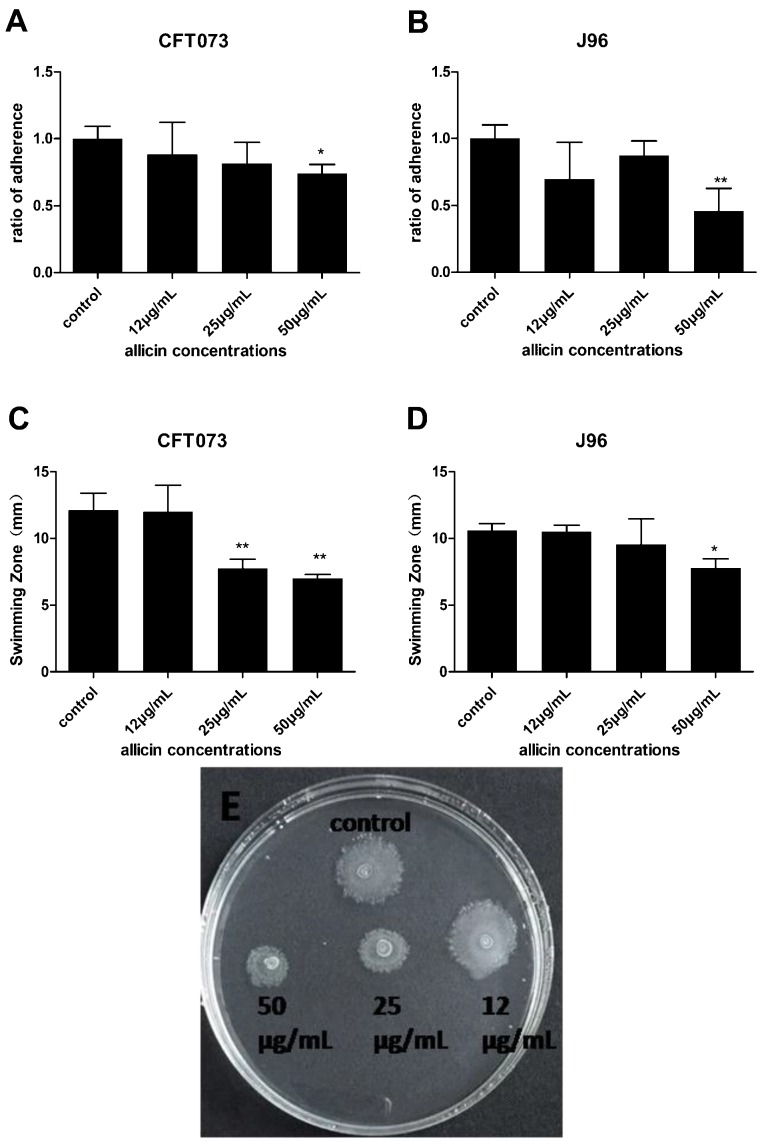
Effect of allicin on UPEC adhesion ability (**A**,**B**) and swimming motility (**C**,**D**). Ratio of adherence = Adherent bacteria of allicin treatment/Adherent bacteria of control. * and ** show statistically significant differences in values (*p* < 0.05 and *p* < 0.01, respectively) compared to the control. Representative image of UPEC CFT073 swimming zone in the absence and presence of allicin was presented (**E**).

UPEC adheres to uroepithelial cells or catheter surfaces to form biofilms [3]. Motility enables UPEC to migrate to a new area facilitating biofilm expansion [3]. Due to their important roles in biofilm formation, UPEC adhesion ability and swimming motility were evaluated in the presence or absence of allicin. As shown in Figure 5A,B, 50 µg/mL allicin decreased the attachment of both CFT073 and J96 in T24 cells, maximal 26% and 54%, respectively. Swimming motility of UPEC was assessed in the presence and absence of allicin by measuring the diameters of swimming zone. UPEC CFT073 swimming motility was drastically decreased in the presence of both 25 and 50 µg/mL of allicin (Figure 5C). UPEC J96 swimming motility was also decreased in the presence of 50 µg/mL of allicin (Figure 5D). Thus, allicin can inhibit UPEC adhesion ability and their swimming motility, which affect their biofilm formation.

### 2.5. Downregulation of fimH in the Presence of Allicin

**Figure 6:**
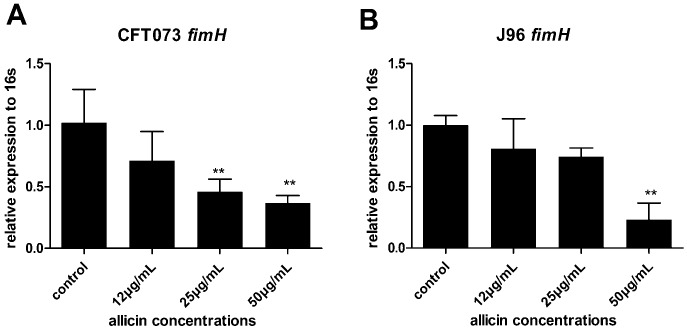
Relative transcript levels of UPEC CFT073 (**A**) and J96 (**B**) type 1 pili adhesin gene *fimH* were assessed by real-time quantitative polymerase chain reaction (RT-qPCR) with 16 s as an internal control relative to the untreated control set at 1. Bars indicate means ± SD of three independent experiments, ** *p* < 0.01.

Type 1 pili adhesin FimH recognizes the glycoprotein uroplakin of bladder epithelial cells thereby mediates UPEC attachment in the bladder epithelium [15]. Thus, the effect of allicin on the expression of UPEC type 1 pili adhesin gene *fimH* was investigated by RT-qPCR. 50 µg/mL of allicin decreased 64% and 77% *fimH* expression of CFT073 and J96, respectively. 25 µg/mL of allicin decreased 54% of fimH expression in CFT073 (Figure 6A,B). Our result suggests that downregulating of *fimH* of UPEC CFT073 and J96 by allicin decreases UPEC adhesion ability.

### 2.6. Docking Analysis of Allicin and FimH

**Figure 7:**
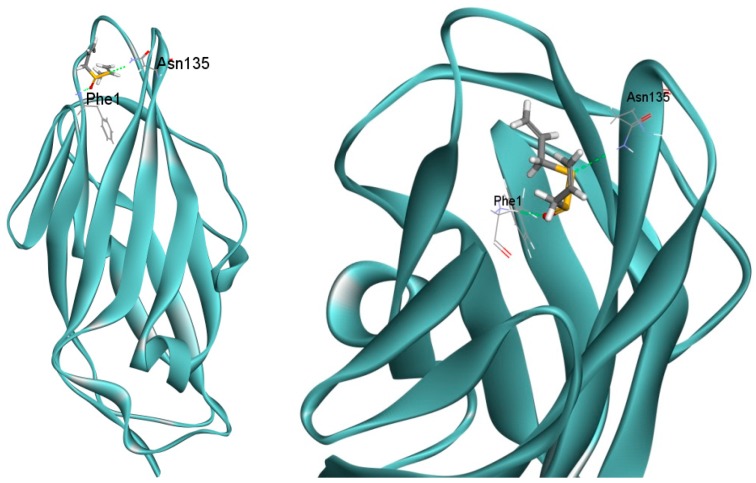
Binding mode of allicin in the FimH lectin domain crystal structure (4BUQ) in complex with heptyl α-d-mannopyrannoside and their main interactions. Allicin was docked into the FimH crystal structure in complex with heptyl α-d-mannopyrannoside (4BUQ). The first pose of 10 docking runs is represented in sticks, with interacting side chains represented by lines.

Previous study showed that α-mannopyranosides can inhibit UPEC adhesion ability by interacting with FimH [16]. In order to predict the binding mode of allicin with FimH protein, molecular docking was performed by docking allicin into the FimH lectin domain crystal structure (4BUQ) in complex with heptyl α-d-mannopyrannosid. 10 docking poses of allicin were located within the binding pocket of heptyl α-d-mannopyrannoside in the crystal structure of FimH (4BUQ). The first pose that has the highest gold score (34.4) formed hydrogen bonds with Phe1 and Asn135 (Figure 7). The result suggests that allicin might inhibit UPEC adhesion ability through interacting with FimH.

### 2.7. Allicin Reduces the Expression of UvrY and Increases the Expression of CsrA in UPEC CFT073

**Figure 8:**
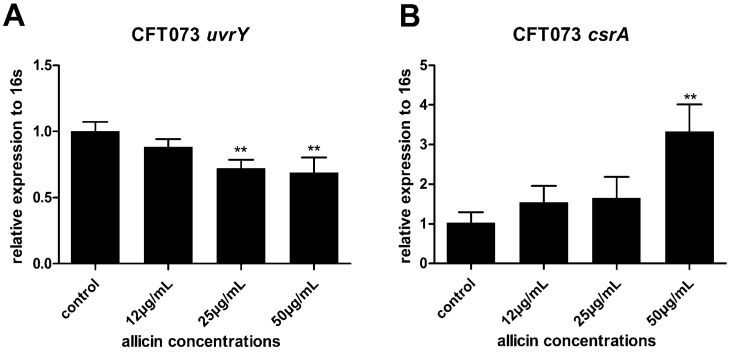
Relative transcript levels of UPEC CFT073 BarA-UvrY gene *uvrY* (**A**) and its downstream global RNA binding protein gene *csrA* (**B**) were assessed by RT-qPCR with 16s as an internal control relative to the untreated control set at 1. Bars indicate means ± SD for three independent experiments, ** *p* < 0.01.

The TCS cognate response regulator UvrY plays a critical role in CFT073 biofilm formation. Its downstream protein CsrA serves as both a repressor of biofilm formation and an activator of biofilm dispersal [17]. UvrY facilitates the biofilm development while the over-expression of CsrA leads to inhibition of biofilm formation in CFT073 [4]. Thus the effects of allicin on the expression of UvrY and CsrA in UPEC CFT073 were investigated via RT-qPCR. Expression of UvrY was decreased approximately 32% while CsrA was increased by 3.3 fold in the presence of 50 µg/mL of allicin (Figure 8A,B), indicating that allicin might affect UPEC biofilm via the BarA-UvrY-CsrA pathway.

## 3. Discussion

Biofilms are the basis of many persistent diseases [18]. Adhesion ability and motility are necessary for biofilm establishment [3] and they are also important for maintaining virulence and pathogenicity of UPEC. Recently, persister cells were proved to be associated with biofilms, causing severe multidrug tolerance [19]. Therefore, it is important to carry out studies on novel strategies against bacterial biofilm. Natural products or food-derived agents are attracting great attention for their anti-biofilm abilities [20,21]. Garlic-derived compounds, such as ajoene and allicin, have shown great antibacterial potential. Ajoene can inhibit genes involved in pathogenicity controlled by quorum sensing [22]. In this study, we investigated another garlic-derived antimicrobial agent, allicin, for its ability to affect the biofilm formation and dispersal, as well as adhesion ability and swimming motility of UPEC CFT073 and J96 and the possible molecular mechanisms. Allicin, at the concentrations we used (12, 25 and 50 µg/mL) exhibited no effect on UPEC growth but decreased biofilm formation. The results are in agreement with previous studies wherein allicin decreased biofilm formation of *Proteus mirabilis* [23] and *Pseudomonas aeruginosa* [24]. Moreover, a significant established biofilm dispersal was observed under the treatment of allicin. Allicin rapidly broke up the biofilms of UPEC within 1 h. Therefore, allicin may aid to release the persisters shielded in biofilm so that antibiotics are capable to kill these dormant cells, which are likely to be responsible for urinary tract infections [19].

It is noteworthy that allicin decreased the attachment of UPEC in human bladder epithelial cell lines T24. As we know, initial attachment of UPEC to the bladder not only aids to establish biofilm [3], but also results in colonization and invasion to host cells [25]. It is clear that one of the most efficient way to prevent UTI is to reduce the attachment of UPEC. In order to overcome hurdles like host immune response or urine flow, UPEC has evolved a variety of virulence factors to adhere tightly to urothelial epithelial cells [26]. Among them, fimbriae are complex surface structures that mediate adherence of bacteria to host epithelial receptors [15]. UPEC CFT073 encodes distinct fimbrial gene clusters, including type 1, P, S, and M fimbriae [27,28]. Type 1 pili adhesin FimH recognizes the glycoprotein uroplakin Ia/IIIa and α3/β1 integrin of bladder epithelial cells and thereby mediates UPEC attachment and invasion [15]. Neutralization of FimH or disruption of its encoding gene impaired the ability of UPEC to colonize the bladder epithelium [29] and the agent downregulated fimH gene showed an inhibitory effect on UPEC attachment and invasion in T24 cells [20]. RT-qPCR study showed that the expression level of *fimH* was significantly decreased in the presence of 50 µg/mL allicin, indicating the inhibitory effect of allicin on UPEC attachment in T24 cells was probably due to its ability to decrease the expression of *fimH* gene. Furthermore, docking results suggested that allicin formed hydrogen bonds with FimH Phe1 and Asn135, which have also been shown to be the binding site of a butyl-α-d-mannoside with FimH [30]. Previous studies have shown that butyl-α-d-mannoside are located within the lectine domain of FimH and has higher affinity for FimH (*K*d = 0.15 µM) than mannose (*K*d = 2.3 µM) [30]. Additionally, α-mannopyranosides can inhibit the adhering process of UPEC to the mannosylated receptors by interacting with FimH [16]. Interacting with the same residues of allicin with butyl α-d-mannoside indicates that the bacterial antiadhesive function could also result from the binding with FimH.

Another observation was the ability of allicin to affect UPEC swimming motility. The flagellum-mediated motility contributes to bacterial swimming and their movement along surfaces, promoting the spread of infection [31]. Furthermore, motility may help the bacteria within a developing biofilm to move along, facilitating spread of the biofilm [3]. Thus, allicin can potentially hamper the dissemination of UPEC infection. Taken together, allicin may affect UPEC biofilm formation by blocking the initial attachment of UPEC to surfaces, and decrease the motility ability to prevent the spread of biofilm.

Earlier study has shown that the BarA/UvrY/CsrA pathway is associated with UPEC CFT073 biofilm formation [4]. *uvrY* controls transcription of the genes, which encode the major fimbrial subunit of type 1 and P fimbriae in CFT073. Mutation in *uvrY* influences transcription of adhesins in CFT073 [4]. uvrY also affects expression of the master regulator, *flhC* and *flhD*, which contribute to flagellum biogenesis [32]. Thus, uvrY might be a key regulator in UPEC biofilm formation. Global RNA binding protein csrA regulates several virulence genes in *E. coli.* Over-expression of *csrA* leads to inhibition of biofilm formation in CFT073 [4] and *E. coli* K12 [17]. CsrA of *E. coli* K12 also serves as an activator of biofilm dispersal [33]. Based on these results, we investigated the relationship between allicin and the BarA/UvrY/CsrA pathway. It was confirmed by RT-qPCR that allicin can downregulate *uvrY* and upregulate *csrA* of CFT073. It is necessary to conduct experiments to investigate the effect of allicin on uvrY/csrA mutant to further confirm our thoughts. The mucoid phenotype we observed by SEM might be due to the over-expression of the exopolysaccharides [4], indicating that allicin might induce other related metabolic genes, such as exopolysaccharide biosynthetic genes, which need further study.

## 4. Materials and Methods

### 4.1. Allicin Preparation

The allicin was synthesized by oxidation of diallyldisulfide with magnesium monoperoxyphthalate using tetrabutyl ammonium hydrogensulfate as a phase transfer catalyst (Figure 1). Purification of allicin was made by preparative thin layer chromatography (TLC) (Silica Gel 60 F254 coated Glass-Backed TLC plates, 92/8 hexane/isopropanol as mobile phase). The band containing allicin (Rf 0.3) appeared clearly under 254 nm light and was collected and extracted with dichloromethane and concentrated in vacuum. The oily residue was dissolved in water and stored at −70 °C. For details on synthesis and analytical methods, see [33].

### 4.2. Bacterial Growth and Determination of MIC

UPEC CFT073 (ATCC 700928) and J96 (ATCC 700336), as well as *E. coli* standard strain (ATCC 25922), were grown in the presence or absence of allicin. For the determination of MIC, according to the recommendation of Clinical and Laboratory Standards Institute [34], the bacteria were grown overnight to log-phase in Luria-Bertani (LB) broth with shaking (170 rpm) at 37 °C and diluted with sterile phosphate buffer saline (PBS) to a concentration of cells equal to 0.5 on the McFarland scale (1 × 10^8^ CFU/mL). Then, Mueller-Hinton broth (MHB) was added to obtain a final inoculum concentration of 2 × 10^5^ CFU/mL. Subsequently, 50 µL of bacterial suspension and 50 µL of two fold serial allicin dilution (at concentrations ranged from 12 to 200 µg/mL) in MHB or 50 µL MHB without allicin were added into 96-well polystyrene microtiter plates and the absorbance of each well was read at 600 nm. The plate was incubated at 37 °C for 24 h and the absorbance was read again at the same wavelength. All experiments were performed in triplicate. The MIC of allicin was defined as the lowest concentration that completely inhibited the bacterial growth.

### 4.3. Biofilm Assays

UPEC CFT073 and J96 were grown in LB broth overnight (37 °C, 170 rpm) and the culture was diluted to a concentration of 2 × 10^7^ CFU/mL in LB broth with sub-MICs of allicin or in LB broth only. Then, 200 µL of aliquots were loaded into a 96-well polystyrene microtiter plate and the plate was incubated for 24 h at 37 °C to allow biofilm formation. After the incubation, the wells were washed twice with PBS to remove non-adherent bacteria. As described elsewhere, the biofilm formation was quantified using the crystal violet assay [35]. Briefly, wells were gently washed with deionized water (DI) water and air dried for 10 min. Then, 200 µL of 0.1% crystal violet in PBS was added to each well and the plate was incubated at 37 °C for 15 min. Wells were washed again with DI water, air dried and 200 µL of 95% ethanol was added. The crystal violet bound to the biofilm was solubilized in the ethanol solution and the OD at 595 nm was measured to estimate the biofilm formed. In order to decrease potential bias, biofilm level was normalized to the level of bacterial growth (determined previously). Biofilm dispersal assay was also performed. As described above, UPEC were grown in LB broth overnight, diluted in fresh LB broth, and inoculated into a 96-well plate. After the biofilm formed, sub-MICs of allicin in LB broth or fresh LB broth only was added and the plate was incubated in 37 °C for another 1 h. The biofilm was also quantified using the crystal violet assay. The absorbance of control was OD_A_ and the absorbance of allicin treated biofilm was OD_B_. The biofilm dispersal level was defined as (OD_A_ − OD_B_)/OD_A_.

### 4.4. Scanning Electron Microscopy

Scanning electron microscopy (SEM,) was also applied for biofilm formation analysis according to Asadishad et al. [36] with some modifications. UPEC were grown overnight and the culture was diluted in allicin or LB broth as described above. Then, 200 µL of the bacterial suspension was placed on glass cover slips in 96-well plate. The plate was incubated for 24 h to allow biofilm formation. The wells were gently washed with PBS and the adhered biofilm was fixed with 200 µL 2.5% glutaraldehyde. After fixation, the wells were washed with PBS again, taken through a graded ethanol and amyl acetate series, air-dried, and metal coated with gold-palladium in a Model E-1010/E-1020 Hitachi Ion Sputter (Joel, Japan). For morphological characterization, a field-emission scanning electron microscope (SEM) Hitachi S-4800 (Hitachi, Japan) was used.

### 4.5. Cell Culture and Bacterial Adherence Assay

Human uroepithelial cells, T24 (ATCC HTB-4), were purchased from the Cell Bank of the Chinese Academic of Science (Shanghai, China) and cultured in RPMI 1640 medium (Hyclone Thermo Scientific, Beijing, China) with 10% fetal bovine serum (FuMeng Gene Co., Ltd., Shanghai, China), 100 U/mL penicillin, and 100 µg/mL streptomycin (Beijing Solarbio Science and Technology Co., Ltd., Beijing, China) at 37 °C in humidified air with 5% CO_2_. Adherence assay was performed according to a previous method [37]. Briefly, an appropriate number of T24 cells were seeded into a 24-well plate and allowed to grow overnight to 2 × 10^5^ cells each well. The cells were washed with PBS and infected with sub-MICs of allicin-treated UPEC (pre-treated for 2 h, adjusted to the same OD_600_ value) at a multiplicity of infection (MOI) of 100:1 for 2 h. Cells were washed with PBS three times to remove non-adherent bacteria. Subsequently, 200 µL of 0.25% Triton X-100 was added and incubated for 15 min to lyse the cell. Cells were diluted and plated on LB agar to quantify the number of adherent bacteria.

### 4.6. Swimming Motility Assay

Swimming motility assay was performed on soft LB-agar plates containing 0.25% agar. The plates were allowed to dry overnight at 4 °C before use. Overnight culture of UPEC CFT073 and J96 were diluted and treated with allicin for 2 h. Swimming plates were seeded below the agar surface with 2 µL bacterial culture. The plates were incubated at 37 °C for 24 h after which the diameters of swimming zones were measured [14].

### 4.7. Real-Time Quantitative Polymerase Chain Reaction (RT-qPCR)

Total RNA was extracted from sub-MICs of allicin treated UPEC using an EZNA total RNA isolation kit (Omega Bio-Tek, Doraville, CA).RNA concentration and quality were confirmed by using NanoPhotometer P330 (Implen, Munich, Germany). A total of 50 ng of RNA was used for cDNA synthesis by a PrimeScript 1st Strand cDNA Synthesis Kit (TaKaRa, Japan) following the manufacturer’s instructions. To determine the expression of target genes of *fimH*, *uvrY*, and *csrA* relative to the endogenous reference gene (16 s), 2 µL Cdna, 0.4 µL of 10 µM forward and reverse primer mixture, 1.2 µL diethyl pyrocarbonate (DEPC) H_2_O, and 4 µL Maxima SYBR Green qPCR Master Mix were added into a PCR reaction tube to create a total 10 µL reaction mixture and then run on the CFX96 Real-Time PCR Detection System (Bio-Rad, Hercules, CA, USA). Then the PCR thermal cycling program was run at 95 °C for 15 s, at 55.5 °C *(fimH*), 59.0 °C (*uvrY*), and 55.5 °C (*csrA*) for 1 min to get a total of 40 cycles of amplification. Primer sequences are given in Table S1, The relative expression of target genes and housekeeping gene 16s was calculated according to the (2^−∆∆*C*t^) method [38].

### 4.8. Molecular Docking

Possible binding sites of allicin within FimH were predicted by docking allicin into the FimH crystal structure (4BUQ) using the program Genetic Optimization for Ligand Docking (GOLD) (Cambridge Crystallography Data Centre, Cambridge, UK) in its implementation in Accelrys Discovery Studio Client (DS) version 3.1 (Accelrys Software Inc., San Diego, CA, USA). Hydrogen atoms were added to all ligands and the receptor prior to performing to the docking runs. The number of docking runs was set to 10, the “Detect Cavity” and “Early Termination” were set to be “False” to allow for the maximum number of runs. All other parameters were left at their default values. Gold scores, hydrogen bonds, and π-interactions of allicin were analyzed for the first pose of the 10 runs.

### 4.9. Statistical Analysis

Data analysis was performed using SPSS version 17 software (SPSS Inc., Chicago, IL, USA). *p* < 0.05 was considered significant. All experiments were performed at least in triplicate.

## 5. Conclusions

Overall, we have shown that sub-MICs of allicin can decrease UPEC CFT073 and J96 biofilm formation, adhesion ability, swimming motility, and disperse developed biofilm, potentially aiding to the eradication of the infection. These effects may be due to the influence of allicin on type 1 pili adhesion FimH and BarA/UvrY/Csr pathway gene *uvrY* and *csrA*. In conclusion, the results suggested a potentially therapeutic application to UPEC biofilm and associated infections.

## Supplementary Materials

Supplementary materials can be found at http://www.mdpi.com/1422-0067/17/7/979/s1.

## Abbreviations

UPEC	Uropathogenic *Escherichia coli*
UTIs	Urinary tract infections
CAUTIs	Catheter-Associated Urinary Tract Infections
TCSs	Two-component systems
Sub-MICs	sub inhibitory concentrations
