# Supplementary material for: Subinhibitory Concentrations of Allicin Decrease Uropathogenic Escherichia coli (UPEC) Biofilm Formation, Adhesion Ability, and Swimming Motility
Source: Int J Mol Sci. 2016 Jun 29;17(7):979. doi: 10.3390/ijms17070979 (PMC4964365; doi:10.3390/ijms17070979)
Supplement: Supplementary file 1 [file ijms-17-00979-s001.pdf]

Supplementary Materials: Subinhibitory Concentrationsof Allicin Decrease Uropathogenic *Escherichia coli* (UPEC) Biofilm Formation, Adhesion Ability, and Swimming Motility

Xiaolong Yang, Kaihui Sha, Guangya Xu, Hanwen Tian, Xiaoying Wang, Shanze Chen, Yi Wang, Jingyu Li, Junli Chen and Ning Huang

Table S1. Primer sequences.

Genes	Primer Sequences (5′–3′)
16s forward	CAAGGGCACAACCTCCAAAT
16s reverse	GTGTAGCGGTGAAATGCGTAGAG
fimH forward	TTTGCGACAGACCAACAACT
fimH reverse	GACATCACGAGCAGAAGCAT
uvrY forward	TCAGACAAACTGGCAAATGG
uvrY reverse	CTATTCAGGGCAGCGTTACA
csrA forward	CCTGGATACGCTGGTAGAT
csrA reverse	TCGTCGAGTTGGTGAGAC

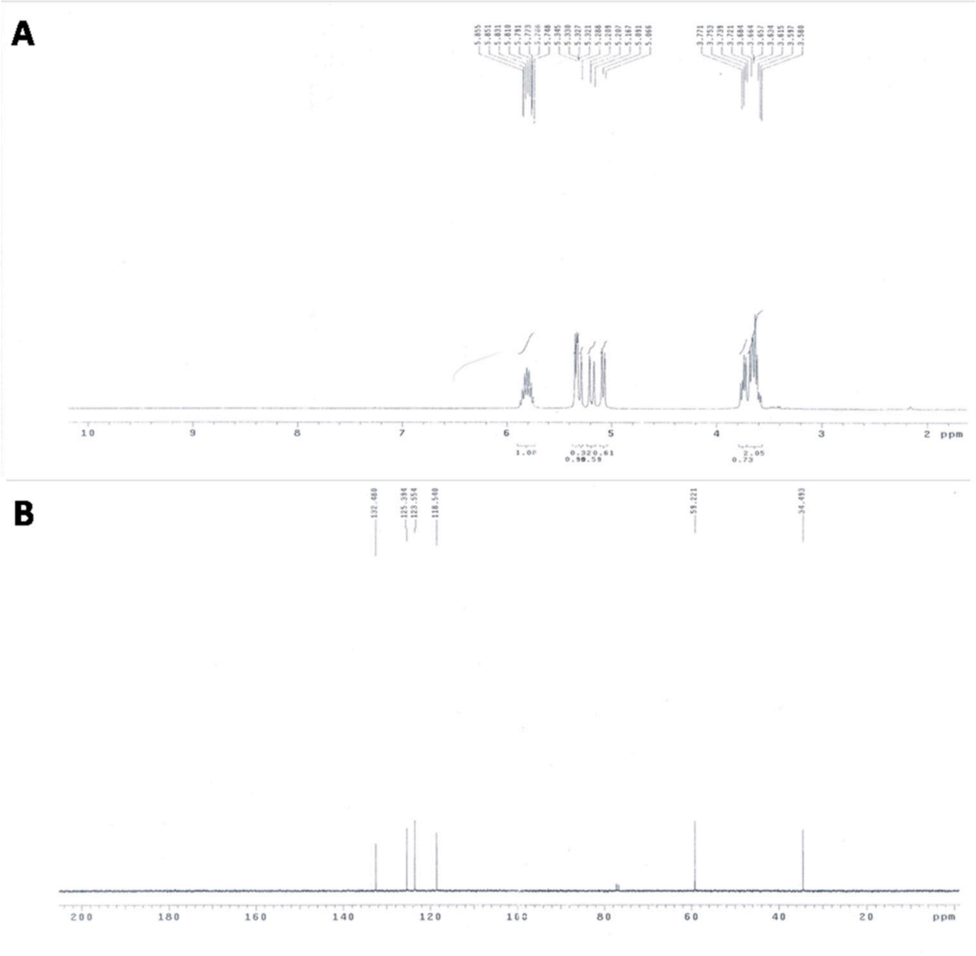

Figure S1. ^{1}H NMR (**A**) and ^{13}C NMR (**B**) spectra of allicin. (**A**) ^{1}H NMR (400 MHz, $CDCl_3$) δ 3.58–3.77 (m, 4H), 5.08 (d, *J* = 10 Hz, 1H), 5.18 (d, *J* = 16 Hz), 5.33 (m, 2H), 5.81 (m, 2H) ppm and (**B**) ^{13}C NMR (150 MHz, $CDCl_3$) δ 34.49, 59.22, 118.54, 123.55, 125.39, 132.48 ppm.

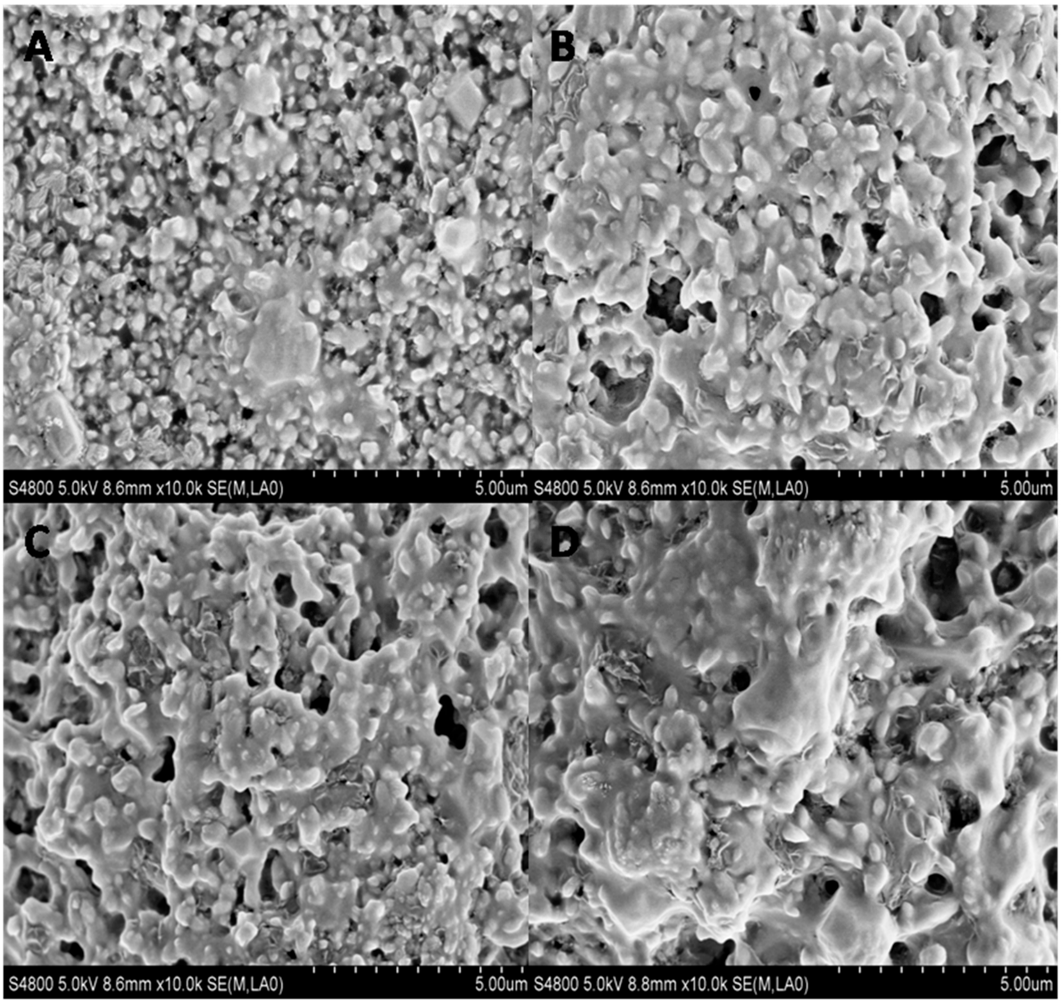

Figure S2. Scanning electron microscopy pictures of growing uropathogenic *Escherichia coli* (UPEC) J96 biofilm. Biofilm architecture was investigated via SEM in the presence or absence of allicin. The images were untreated control (**A**) or treated with 12 μg/mL (**B**), 25 μg/mL (**C**), and 50 μg/mL (**D**) of allicin, respectively.
